# Supplementary material for: In Vivo Study on the Salivary Kinetics of Two Probiotic Strains Delivered via Chewing Gum
Source: Microorganisms. 2025 Mar 24;13(4):721. doi: 10.3390/microorganisms13040721 (PMC12029345; doi:10.3390/microorganisms13040721)

Supplementary material:

- Figure S1. The chewing gum production process.
- Figure S2. Chewing gum used in the study.
- Table S1. Salivary counts (log_{10} CFU/mL) at different time points of the probiotic strains.
- Figure S3. Viable count kinetics of probiotic cells in salivary samples of each subject.

Figure S1. The chewing gum production process.

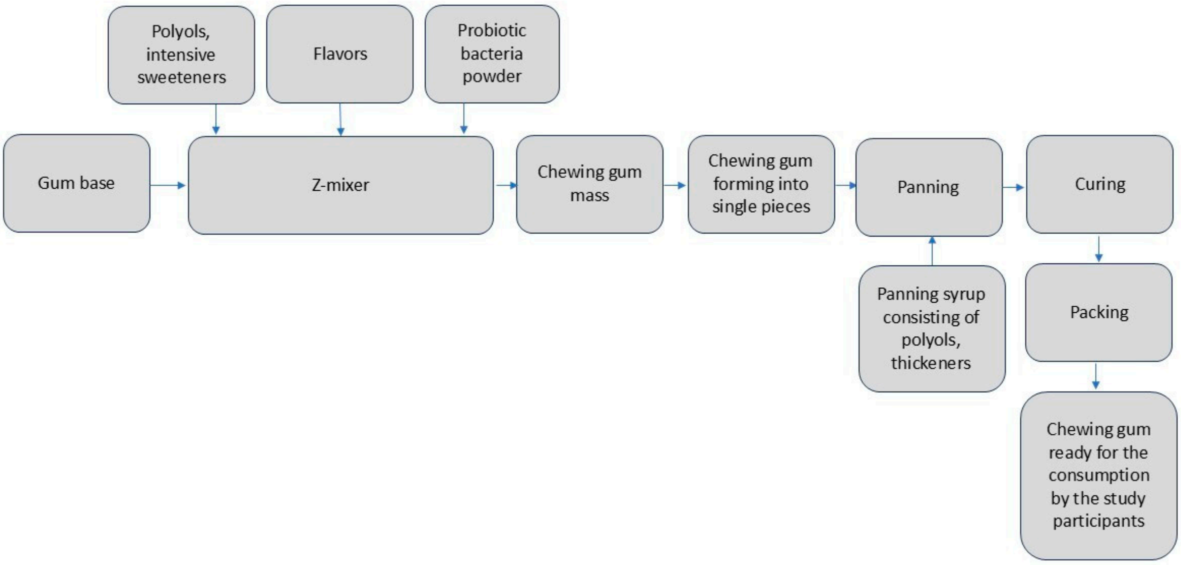

Figure S2. Chewing gum used in the study.

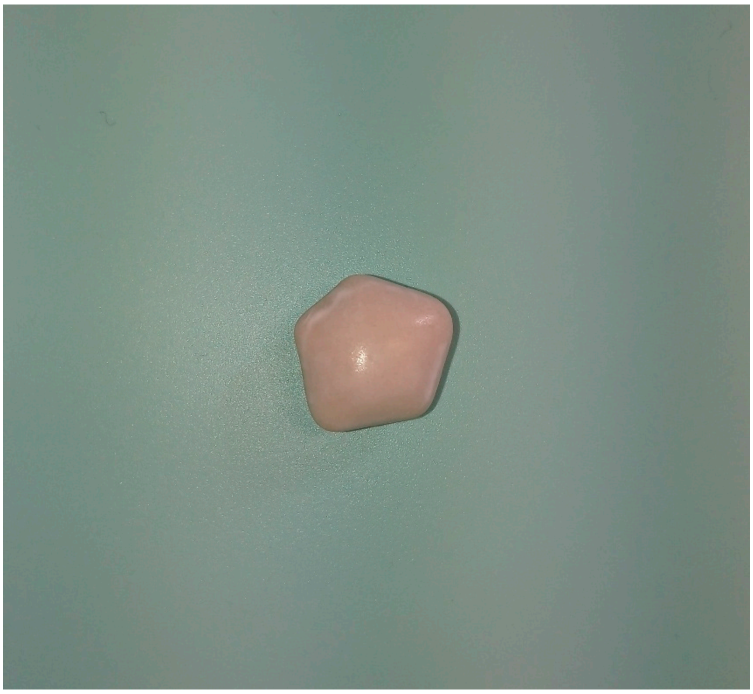

Table S1. Salivary counts (log_{10} CFU/mL) at different time points of the probiotic strains.

Time	Probiotic strain	***H. coagulans* SNZ1969® with spores included (log_{10} CFU/mL)**	***H. coagulans* SNZ1969® pasteurized (log_{10} CFU/mL)**	***H. coagulans* SNZ1969® with spores excluded (log_{10} CFU/mL)**	**LGG® (free form) (log_{10} CFU/mL)**	***L. rhamnosus* GG in micro-encapsulated form (log_{10} CFU/mL)**	**p-value**	
**T_0**	Mean±SD	0.0±0.0	0.0±0.0	0.0±0.0	0.0±0.0	0.0±0.0		
	Median	0.0	0.0	0.0	0.0	0.0		
	Min-Max	0.0; 0.0	0.0; 0.0	0.0; 0.0	0.0; 0.0	0.0; 0.0		
	95% CI	0.0; 0.0	0.0; 0.0	0.0; 0.0	0.0; 0.0	0.0; 0.0		
**T_1**	Mean±SD	6.1±0.5	5.6±0.4	5.8±0.7	5.8±0.3	3.3±0.6	**<0.01**[a*]	**<0.01**[d µ]
	Median	6.3	5.8	6.2	5.8	3.4	0.74[b µ]	0.64[e µ]
	Min-Max	5.4; 6.8	4.9; 6.2	4.7; 6.7	5.3; 6.2	2.0; 4.4	0.92[c µ]	
	95% CI	5.8; 6.5	5.3; 5.9	5.3; 6.4	5.6; 6.0	2.8; 3.7		
**T_2**	Mean±SD	5.8±0.5	5.4±0.1	5.1±1.8	5.1±0.4	2.9±0.7	**<0.01**[a§]	**<0.01**[d µ]
	Median	5.7	5.5	5.4	5.2	2.9	0.43[b µ]	0.52[e µ]
	Min-Max	4.9; 6.5	4.3; 6.0	0.0; 6.3	4.4; 5.7	1.5; 3.9	0.94[c µ]	
	95% CI	5.4; 6.2	5.0; 5.8	3.7; 6.4	4.8; 5.4	2.4; 3.4		
**T_3**	Mean±SD	5.6±0.5	5.1±0.6	4.9±1.7	4.7±0.4	2.7±0.4	**<0.01**[a§]	**<0.01**[d µ]
	Median	5.6	5.1	5.5	4.6	2.7	0.43[b µ]	0.24[e µ]
	Min-Max	4.6; 6.4	4.3; 5.8	0.0; 6.2	4.1; 5.6	1.9; 3.5	0.96[c µ]	
	95% CI	5.2; 5.9	4.7; 5.5	3.6; 6.1	4.4; 5.0	2.4; 3.0		
**T_4**	Mean±SD	3.4±0.6	2.3±1.2	3.3±0.6	4.0±0.7	1.7±1.2	**<0.01**[a§]	**<0.01**[d µ]
	Median	3.6	2.6	3.5	3.9	2.2	1.00[b µ]	0.67[e µ]
	Min-Max	2.5; 4.2	0.0; 3.5	2.5; 4.1	3.0; 5.0	0.0; 3.1	**0.01**[c µ]	
	95% CI	3.0; 3.9	1.3; 3.2	2.9; 3.8	3.5; 4.5	0.8; 2.6		
**T_5**	Mean±SD	2.7±0.4	1.7±1.2	2.3±0.9	3.8±0.9	1.4±1.0	**<0.01**[a*]	**<0.01**[d µ]
	Median	2.7	2.2	2.4	3.9	1.8	0.82[b µ]	0.08[e µ]
	Min-Max	2.1; 3.3	0.0; 3.2	0.0; 3.3	2.3; 4.9	0.0; 2.5	0.71[c µ]	
	95% CI	2.4; 3.0	0.8; 2.6	1.6; 2.9	3.2; 4.5	0.7; 2.2		
**T_6**	Mean±SD	1.5±1.3	1.11±1.12	1.3±1.1	3.3±1.3	1.0±1.0	**<0.01**[a*]	**<0.01**[d µ]
	Median	2.2	1.0	1.5	3.5	1.1	0.99[b µ]	**0.02**[e µ]
	Min-Max	0.0; 3.1	0.0; 2.6	0.0; 2.9	0.0; 4.5	0.0; 2.3	1.00[c µ]	
	95% CI	0.6; 2.5	0.3; 2.0	0.4; 2.1	2.3; 4.2	0.3; 1.7		
**p-value**	T_{all}	**<0.01**[§]	**<0.01**[§]	**<0.01**[§]	**<0.01***	**<0.01**[§]		
	T_1 *vs* T_2 [µ]	0.93	1.00	0.42	0.95	0.76		
	T_2 *vs* T_3 [µ]	0.97	0.99	0.84	0.99	1.00		
	T_3 *vs* T_4 [µ]	**<0.01**	**<0.01**	0.35	0.12	0.10		
	T_4 *vs* T_5 [µ]	0.27	0.81	1.00	0.98	0.42		
	T_5 *vs* T_6 [µ]	**<0.01**	0.70	0.53	0.87	0.52		
**Trend**[¥]	z	-6.20	-5.69	-4.85	-5.02	-5.64		
	p	**<0.01**	**<0.01**	**<0.01**	**<0.01**	**<0.01**		

* Anova; [§] Welch's t-test; [µ] Tukey's range test; [¥] Cuzick's test with rank scores; [a] LGG® (free form) *vs L. rhamnosus* GG in microcapsulated *vs H. coagulans* SNZ1969® with spores included *vs H. coagulans* SNZ1969® with spores excluded *vs H. coagulans* SNZ1969® pasteurized; [b] *H. coagulans* SNZ1969® with spores included *vs H. coagulans* SNZ1969® with spores excluded; [c] *H. coagulans* SNZ1969® with spores excluded *vs H. coagulans* SNZ1969® pasteurized; [d] LGG® (free form) *vs L. rhamnosus* GG in microencapsulated form; [e] LGG® (free form) *vs H. coagulans* SNZ1969® with spores included; T_{all} : T_1 *vs* T_2 *vs* T_3 *vs* T_4 *vs* T_5 *vs* T_6; SD: Standard Deviation; CI: Confidence Interval.

Figure S3. Viable count kinetics of probiotic cells in salivary samples of each subject.

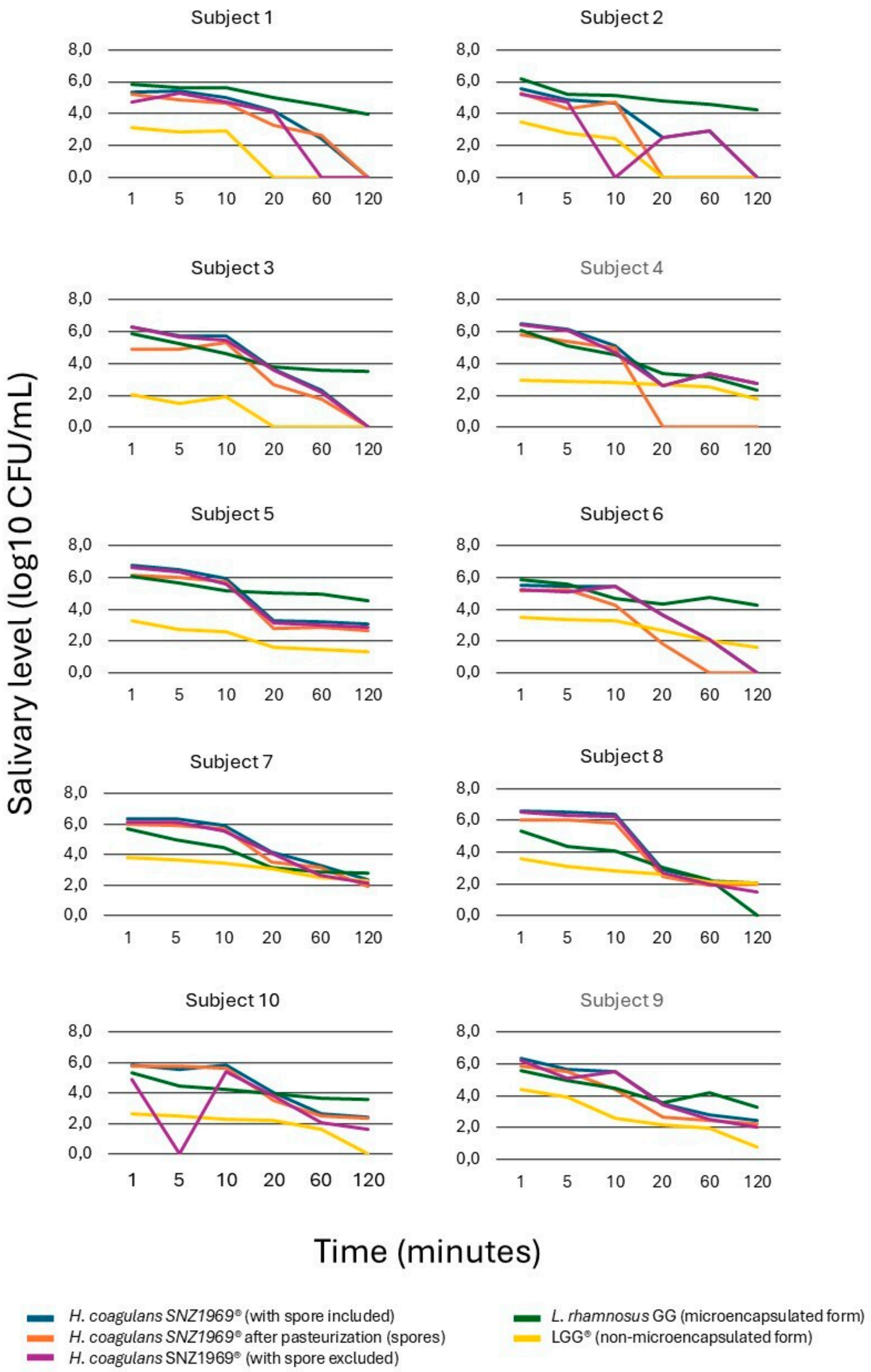

Supplement: Supplementary file 1 [file microorganisms-13-00721-s001.zip › microorganisms-3515252-supplementary.pdf]
